# 3D bioprinting of methacrylated hyaluronic acid (MeHA) hydrogel with intrinsic osteogenicity

**DOI:** 10.1371/journal.pone.0177628

**Published:** 2017-06-06

**Authors:** Michelle T. Poldervaart, Birgit Goversen, Mylene de Ruijter, Anna Abbadessa, Ferry P. W. Melchels, F. Cumhur Öner, Wouter J. A. Dhert, Tina Vermonden, Jacqueline Alblas

**Affiliations:** 1 Department of Orthopaedics, University Medical Center Utrecht, Utrecht, the Netherlands; 2 Department of Pharmaceutics, Utrecht Institute for Pharmaceutical Sciences (UIPS), Utrecht University, Utrecht, the Netherlands; 3 Faculty of Veterinary Medicine, Utrecht University, Utrecht, the Netherlands; Kyoto Daigaku, JAPAN

## Abstract

In bone regenerative medicine there is a need for suitable bone substitutes. Hydrogels have excellent biocompatible and biodegradable characteristics, but their visco-elastic properties limit their applicability, especially with respect to 3D bioprinting. In this study, we modified the naturally occurring extracellular matrix glycosaminoglycan hyaluronic acid (HA), in order to yield photo-crosslinkable hydrogels with increased mechanical stiffness and long-term stability, and with minimal decrease in cytocompatibility. Application of these tailor-made methacrylated hyaluronic acid (MeHA) gels for bone tissue engineering and 3D bioprinting was the subject of investigation. Visco-elastic properties of MeHA gels, measured by rheology and dynamic mechanical analysis, showed that irradiation of the hydrogels with UV light led to increased storage moduli and elastic moduli, indicating increasing gel rigidity. Subsequently, human bone marrow derived mesenchymal stromal cells (MSCs) were incorporated into MeHA hydrogels, and cell viability remained 64.4% after 21 days of culture. Osteogenic differentiation of MSCs occurred spontaneously in hydrogels with high concentrations of MeHA polymer, in absence of additional osteogenic stimuli. Addition of bone morphogenetic protein-2 (BMP-2) to the culture medium further increased osteogenic differentiation, as evidenced by increased matrix mineralisation. MeHA hydrogels demonstrated to be suitable for 3D bioprinting, and were printed into porous and anatomically shaped scaffolds. Taken together, photosensitive MeHA-based hydrogels fulfilled our criteria for cellular bioprinted bone constructs within a narrow window of concentration.

## Introduction

Natural hydrogels are increasingly applied in regenerative medicine, as they can provide scaffolds with unique biocompatible and biodegradable properties [1]. Many of the materials used can be tailored to accommodate various shapes and sizes and functional groups can be incorporated to introduce specific desirable physical and chemical characteristics [2,3]. Due to these properties, hydrogels can be employed as cell-friendly materials that can present signals to guide cellular processes and release proteins or drugs in a controlled fashion [4]. Altogether, this makes hydrogel scaffolds very suitable for tissue engineering purposes, the reason why worldwide research is increasingly focusing on the manufacturing and optimization of hydrogels [5–7].

In order to present cell laden hydrogel scaffolds with a desired shape for tissue engineering, several technologies have been applied, such as molding [8,9], lithography [10], and 3D bioprinting [11]. 3D bioprinting offers the advantage of creating porous constructs with predefined complex architecture, allowing deposition of specific cell types, signaling molecules or biomaterials at predefined regions [12]. This technique allows better mimicry of native tissue organization compared to the other deposition methods mentioned above, for example by addition of specific and predefined vasculature stimulating regions to a bone promoting scaffold.

Hyaluronic acid (HA), [α-1,4-D-glucuronic acid-β-1,3-*N*-acetyl-D-glucosamine]_n_ is a naturally occurring high molecular weight hydrophilic glycosaminoglycan. It is an abundant polymer present in the extracellular matrix where it gives mechanical support [13]. The chemical structure of HA allows easy modification of the (primary) hydroxyl-groups by esterification. Modification with methacrylate groups, followed by addition of a photo initiator leads to polymerization upon UV-exposure, resulting in network formation. Methacrylated HA (MeHA) hydrogels have shown increased rigidity and are more resistant to degradation, compared to non-derivatized HA hydrogels, while maintaining good biocompatibility [14]. Gel stiffness after methacrylation is highly dependent on the degree of substitution (DS, defined as the number of methacrylate groups per 100 disaccharide units), and may be used to steer differentiation of incorporated cells [15]. Methacrylation allows hydrogels to be 3D printed, as scaffolds can be crosslinked with UV light directly after gel deposition to fixate their shape [16]. Osteogenic differentiation of mesenchymal stromal cells (MSCs) was observed during their incorporation in photocrosslinked low molecular weight HA [17]. This finding is promising for the application of 3D printed MeHA scaffolds for the purpose of bone tissue engineering.

MeHA can be obtained using different protocols, with reaction taking place either in organic solvents or aqueous environment [18], yielding various degrees of substitution. Control over the degree of substitution is of major importance to obtain materials with tuneable mechanical properties in a reproducible manner [19]. Therefore, in this study, we apply a protocol that utilizes a mixture of water and *N*,*N*-dimethylformamide (DMF) [20].

The aim of this study was to select a MeHA hydrogel composition for the development of 3D printable scaffolds supporting bone-like tissue formation. Gel properties such as visco-elastic behaviour, swelling and degradability *in vitro*, viability and osteogenic differentiation of encapsulated primary cells, as well as 3D bioprintability of the gel were the main subjects of investigation.

## Material and methods

### Synthesis of MeHA

Hyaluronic acid sodium salt (HA, M_W_ = ~1.7*10^6^ g/mol, from *Strept*. *Equi*, Sigma-Aldrich, Zwijndrecht, the Netherlands) was methacrylated following an adapted protocol by Hachet et al [20]. In short, 2.0 g HA (corresponding to 5.0 mmol of disaccharide units) was dissolved in 150 ml of reverse osmosis (RO) water and stirred overnight at RT for complete dissolution. Subsequently, DMF (Biosolve, Valkenswaard, the Netherlands) was added drop wise to obtain a water/DMF ratio of 3/2 (v/v). Methacrylic anhydride (MA, Sigma-Aldrich) was added using a molar ratio HA disaccharide unit/MA ranging from 1/1.5 to 1/3, while maintaining the pH at 8 to 9 using 0.5 M NaOH. After overnight stirring, the mixture was diluted with RO-water (final water/DMF ratio of 10/1 (v/v)) and sodium chloride (NaCl, Merck, Darmstadt, Germany) was added to achieve a final concentration of 0.5 M. After this, the solution was transferred to a dialysis membrane (M_W_ 12–14 kDa cut-off, Medicell, London, UK) and dialyzed for 5 days at 4°C against RO water. The remaining HA solution was lyophilized overnight and kept at -20°C until characterization and use.

### High performance liquid chromatography (HPLC) and nuclear magnetic resonance (NMR)

For ^1^H-NMR, a solution was prepared (6% (w/w)) of MeHA polymer in D_2_O and analyzed on a 300 mHz Gemini Spectrometer (Varian, Palo Alto, CA). All samples were measured with a relaxation delay of 0.6 seconds for 512 scans. The degree of methacrylate substitution was calculated by comparing the integrals of the peaks of the methacrylate groups at 1.9, 5.7 and 6.1 ppm relative to the integrals originating from the protons of HA.

For the HPLC measurements, 15 mg of MeHA was dissolved in 10 ml of 0.02 M NaOH solution, and incubated at 37°C for 30 minutes to ensure complete hydrolysis of the ester bonds. After that, 2 ml of 2 M acetic acid (Merck) was added to acidify the solution. Samples were filtered over 0.2 μm filters and injected onto a Sunfire RP-18 column (Lichrospher, Darmstadt, Germany). Samples were analyzed using a HPLC Waters 2695 system equipped with an UV detector model 2487 (λ = 210 nm, Waters Inc., Dublin, Ireland). The mobile phase consisted of a mixture of acetonitrile (Actu-All Chemicals, Oss, the Netherlands) and water (ratio: 15/85) adjusted to pH 2 with perchloric acid (HClO_4_, 70%, Sigma-Aldrich) and a flow rate of 1 ml/min was used. A calibration curve was obtained by injection of methacrylic acid solutions in eluent with a concentration range of 0 to 160 μg/ml. Empower Pro software (Waters) was used to calculate the concentration of methacrylic acid.

### Experimental design

MeHA was dissolved in a concentration range in alpha minimum essential medium (α-MEM, Gibco, Breda, The Netherlands), and stirred overnight. A 1% (w/v) stock solution of photoinitiator (Irgacure 2959, Ciba Specialty Chemicals, Basel, Switzerland) was prepared at 90°C, cooled down and then added to the polymer solution in order to achieve a final concentration of 0.1%. The resulting polymer concentrations ranged from 1% to 3% (w/v). The polymer solutions and their UV-crosslinked products were analyzed in terms of mechanical strength, biodegradability, biocompatibility, 3D printability and the ability of MSCs to differentiate when incorporated into the gels.

### Hydrogel swelling and degradation

Polymer solutions (1–3% (w/v)) were photopolymerized for 10 minutes at 365 nm and 3 mW/cm^2^ (1800 mJ/cm^2^) in a UVP CL-1000L crosslinker (UVP, Upland, Ca, USA) using custom made Teflon moulds (50x4x2 mm), then cut and transferred to pre-weighed microtubes and weighed (W_0_). The gels were then immersed in 5 ml PBS (n = 5) or PBS supplemented with 2.6 U/ml hyaluronidase (type II, H2126, Sigma) (n = 5) in accordance to endogenous enzymatic circumstances [21]. The buffer was discarded at multiple time points, and gels were weighed (W_wet_), lyophilized and weighed again (W_dry_). Swelling was calculated as W_wet_ /W_0_, degradation as W_dry_/W_0_.

### Rheological analysis

Rheological analysis of the MeHA hydrogels was performed on an ARG2 rheometer with UV curing accessory (TA Instruments, Etten-Leur) using a 20 mm parallel plate UHP steel. 120 μl of gel was placed between the two plates and analysed at 37°C under oscillation mode using 1% strain and a frequency of 1 Hz. Exposure to UV-light (365 nm) occurred after 5 minutes using a bluepoint 4 UV-lamp (Honle UV technology) at 40 mW/cm^2^ (1200 mJ/cm^2^) and lasted for 5 minutes. G’ (storage modulus) and G” (loss modulus) were monitored at RT for 15 minutes. Moduli and tan delta were calculated as the mean of values measured after a plateau level was achieved.

### Dynamic mechanical analysis

Hydrogel discs of 200 μl were obtained (1–3% (w/v)) using a syringe with a diameter of 8 mm and a height of ± 3 mm as a mould, by UV-photopolymerization for 10 minutes at 6 mW/cm^2^. Compression tests were performed in triplicate on a 2980 DMA (TA Instruments) with a ramp force from 0.1–1 N for 10 minutes. The elastic modulus was calculated as the slope of the stress-strain curve that was obtained from the compression test.

### Multipotent stromal cells (MSC)

Human bone marrow-derived MSCs were isolated from bone marrow aspirates, acquired during orthopedic surgery of patients after written informed consent. Acquiring the bone marrow was approved by the Institutional Medical Ethical Review Committee (METC, approval number 08-001K).

The mononuclear fraction was isolated using Ficoll density gradient centrifugation. The MSCs were isolated by their adherence to tissue culture plastic, and expanded in αMEM, supplemented with 10% (v/v) fetal calf serum (Cambrex, Charles City, IA, USA), 100 U/ml penicillin, 100 μg/ml streptomycin, 0.2 mM L-ascorbic acid-2-phosphate (AsAP, Sigma-Aldrich) and 1 ng/ml basic fibroblast growth factor (bFGF, R &D Systems, Minneapolis, MN, USA). Cells were cultured in a humidified incubator at 37°C and 5% CO_2_.

### MSC survival in MeHA hydrogels

Human bone marrow-derived MSCs were incorporated into 1–3% (w/v) MeHA hydrogels at a density of 2x10^6^ cells/ml, and photopolymerized in UV mold chambers at 1800 mJ/cm^2^ (10 minutes, 3 mW/cm^2^). The cell-laden gels were subsequently cultured in expansion medium (control) or expansion medium supplemented with 1 μg/ml bone morphogenetic protein 2 (BMP-2) until analysis. To quantify MSC viability, a LIVE/DEAD Viability Assay (Molecular ProbesMP03224, Eugene, Oregon, USA) was performed according to the manufacturer’s protocol. Samples were examined in triplicate using an Olympus BX51 light microscope with excitation/emission filters set at 488/530 nm to observe living (green) cells and at 530/580 nm to detect dead (red) cells. Three pictures were taken at random locations throughout each sample. Live and dead cells were counted automatically using Image J software [22] with identical settings after 1 and 21 days of culture in MSC expansion medium.

### MSC osteogenic differentiation in MeHA hydrogels

Human bone marrow-derived MSCs were incorporated into 1–3% (w/v) MeHA hydrogels at a density of 2.0x10^6^ cells/ml, photopolymerized in UV mold chambers at 1800 mJ/cm^2^. The cell-laden gels were subsequently cultured in expansion medium (control) or expansion medium supplemented with BMP-2 until analysis. After 21 days, osteogenic differentiation was quantified using a calcium assay kit (DICA-500, QuantiChrom, BioAssay Systems, Hayward, CA, USA) measuring calcium deposition by the MSCs per mg of scaffold. Additionally, Alizarin red staining was performed on these scaffolds to visualize scaffold calcification. For this, a 2% Alizarin Red S (Fluka 5600) solution in water was used, with the pH set to 4.7 by addition of 0.5% ammonium hydroxide (NH_4_OH in H_2_O, Merck). The gels were soaked in this solution for 1 minute and then thoroughly washed with demi water.

### 3D bioprinting

MeHA was dissolved in α-MEM as described above to yield 1–3% (w/v) hydrogels, including 0.1% photoinitiator Irgacure 2959. Subsequently, these hydrogels were 3D bioprinted using the Bioscaffolder dispensing system (SYS+ENG, Gladbeck, Germany) [23]. Scaffold architecture was designed as either porous cube or non-porous human L3 vertebrae shapes (acquired from a CT scan) and converted to computer-aided design (CAD) files. CAD files were then combined with specific material settings to a numerical control (NC) code, which directs XYZ controller of the 3D printer [24]. Porous scaffolds measuring 20x20x3 mm, and lumbar scaffolds measuring 20x25x1 mm were 3D bioprinted under sterile conditions in a laminar flow-cabinet, using a 25 G needle, with a strand thickness of 0.2 mm and strand distances of 1 mm (porous), or 0.2 mm (non-porous). After 3D bioprinting, the scaffolds were UV irradiated with a Superlite S-UV 2001 AV lamp (Lumatec, Munchen, Germany) at 1800 mJ/cm^2^ to ensure crosslinking.

### Statistical analysis

Data are represented as mean ± standard deviation, and analyzed using an ANOVA test with post hoc Bonferroni correction. Differences were considered statistically significant when p<0.05.

## Results

### Synthesis and characterization of MeHA

**Fig 1:**
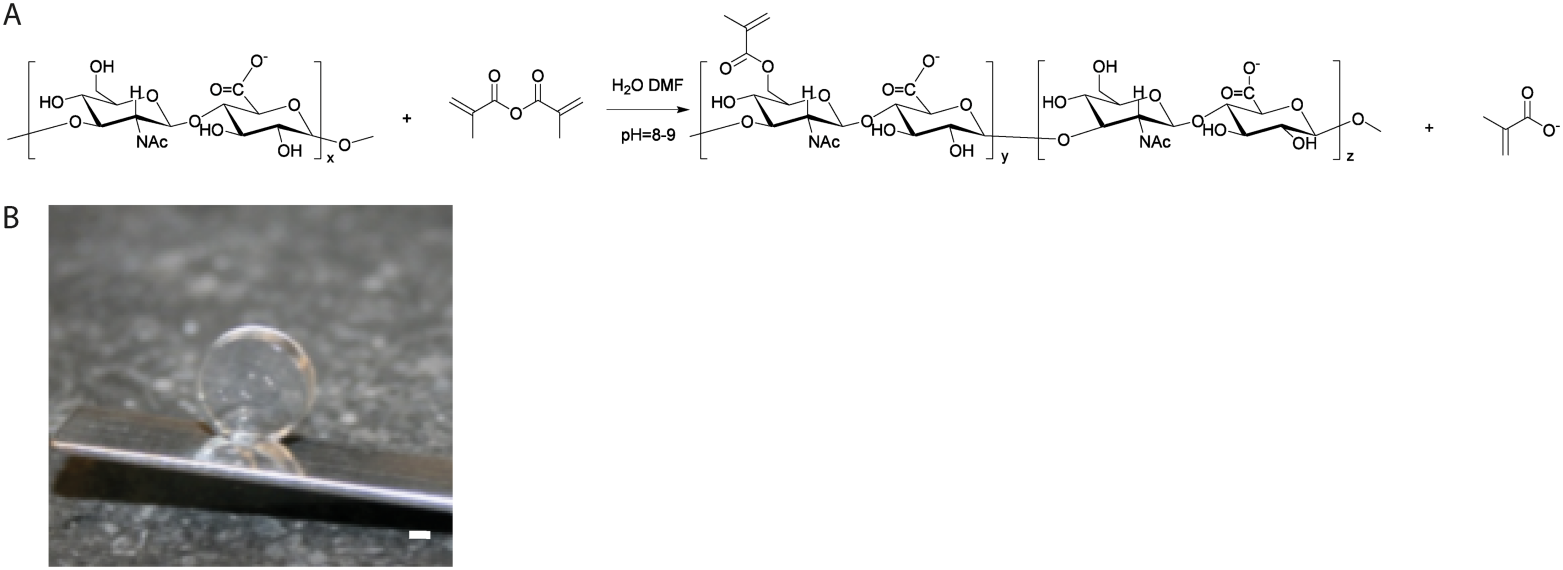
Schematic representation for the methacrylation reaction of HA and photograph of cross-linked MeHA gel. A. HA reacted with methacrylic anhydride in an aqueous environment in presence of DMF, yielding the photo-crosslinkable MeHA. B. Hydrogel disc prepared from 3% MeHA (DS = 5%). Scale bar represents 1 mm.

MeHA was acquired by methacrylation of HA (Fig 1A). Methacrylation was visible in NMR with peaks at 1.9, 5.7 and 6.1 ppm, but accurate quantification was difficult due to overlapping peaks. Therefore, HPLC of degraded samples at high pH was performed to quantify hydrolysed methacrylic acid. Variations in HA disaccharide unit/MA ratios and batch sizes led to DS values of 6.3 ± 2.8. Polymer batches with a DS between 5 and 7% were selected for further experiments, since these yielded hydrogels with visco-elastic properties that allow handling with a pipette, a necessity for the experiments that involve cell-incorporation, moulding and 3D bioprinting (Fig 1B).

Effectivity of UV-induced MeHA crosslinking over time was assessed by rheometry. After UV exposure, a sharp increase in storage modulus was observed at all polymer concentrations tested, and this increased with higher concentrations of MeHA polymer within the hydrogel (Table 1). This increased storage modulus indicated effective intermolecular crosslink formation by photopolymerization of HA chains. The loss modulus (viscosity) increased negligibly during this time. Tan delta values indicated that after gelation the MeHA hydrogels were more elastic than viscous (Tan δ <1). Subsequently, mechanical properties of the hydrogels were measured by dynamic mechanical analysis (DMA). Elastic moduli increased with increasing concentrations, indicating higher material stiffness (Table 1).

**Table 1:** MeHA hydrogel characteristics before and after application of UV irradiation.

*(w/v)* *%*	*Storage modulus*^a^ *G’ (Pa)* *before UV*	*Storage modulus*^a^ *G’(Pa)* *after UV*	*Loss modulus*^a^ *G” (Pa)* *after UV*	*Elastic modulus E*^b^ *(kPa)* *after UV*
*1*	*5 ± 0*.*5*	*170 ± 63*	*7 ± 2*	*1*.*3 ± 0*.*1*
*1*.*5*	*15 ± 2*	*205 ± 66*	*7 ± 6*	*4*.*1 ± 0*.*5*
*2*	*26 ± 9*	*374 ± 197*	*8 ± 4*	*6*.*3 ± 1*.*2*
*2*.*5*	*66 ± 4*	*766 ± 150*	*11 ± 2*	*6*.*8 ± 1*.*2*
*3*	*200 ± 15*	*2602 ± 199*	*22 ± 4*	*10*.*6 ± 0*.*1*

^a^ Measured with rheometry,

^b^ measured with dynamic mechanical analysis (DMA). Data are represented as mean ± SD of n = 3.

### Hydrogel swelling and degradation

**Fig 2:**
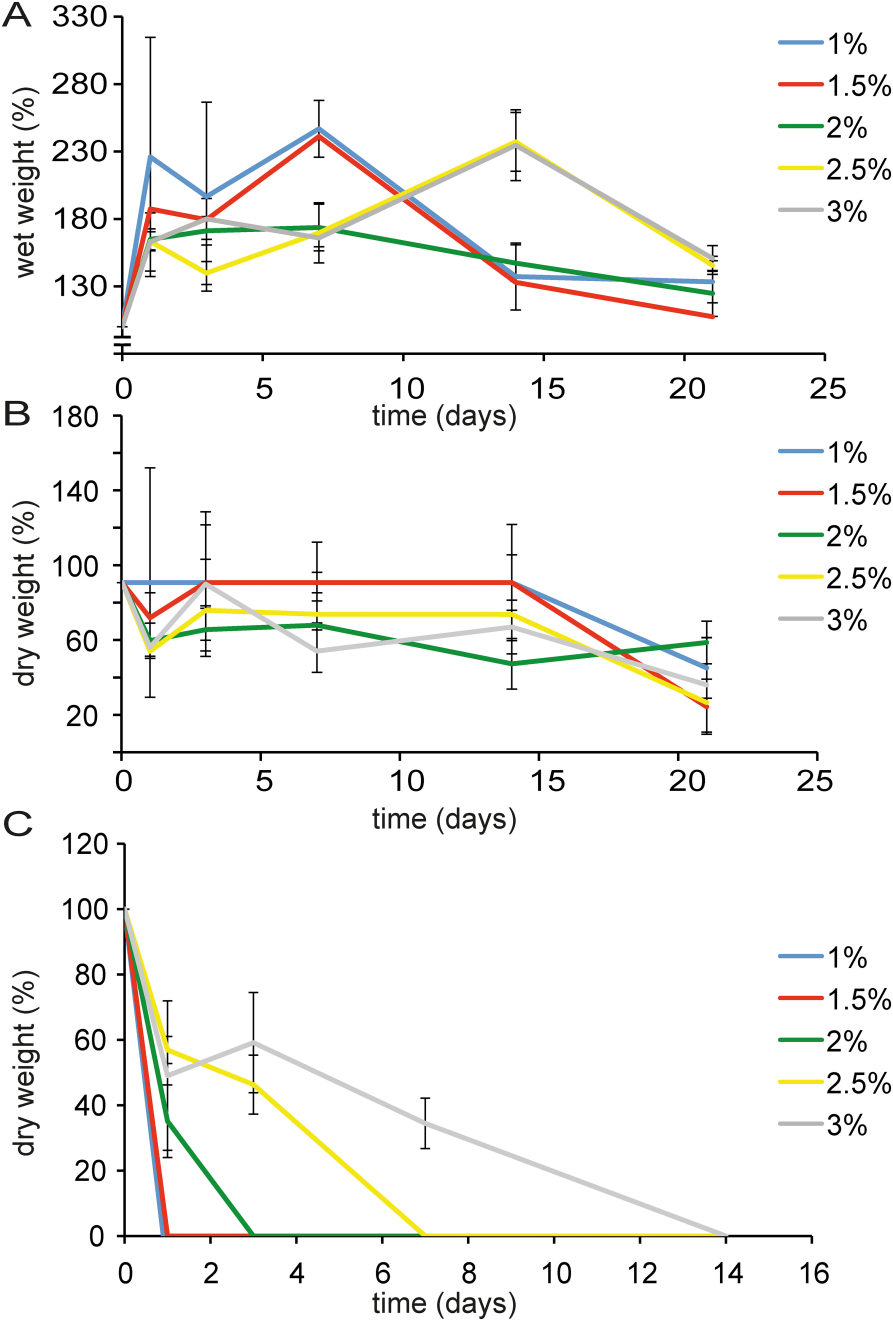
MeHA hydrogel swelling and degradation as a function of gel concentration. A. Increase of hydrogel wet weight (swelling) during 3 weeks incubation at 37°C, which was most pronounced at lower MeHA concentrations. B. Decrease of hydrogel dry weight during incubation. C. Decrease of hydrogel dry weight followed in time in the presence of hyaluronidase. Gel degradation was slower with increasing MeHA concentration. Data presented as mean ± SD, n = 5 for all measurements.

The wet weight increase of hydrogels as function of time is presented in Fig 2A. The swelling (wet weight increase) in the initial 24 hours was largest for the lowest concentration hydrogel (1% w/v) and decreased in the more concentrated hydrogels. Degradation of the gels occurred fastest in the lower percentage gels, hereby lowering their ability retain water, reflected in their decreasing wet weight. In PBS without enzymatic supplements, the dry weight loss became evident after 14 days in a similar way for almost all the gels tested (Fig 2B). When hyaluronidase was added at physiological concentrations, the 1 and 1.5% (w/v) gels disintegrated within 24 hours. The more concentrated gels lasted longer. All gels enzymatically degraded within 2 weeks of incubation (Fig 2C).

### MSC survival in MeHA hydrogels

**Fig 3:**
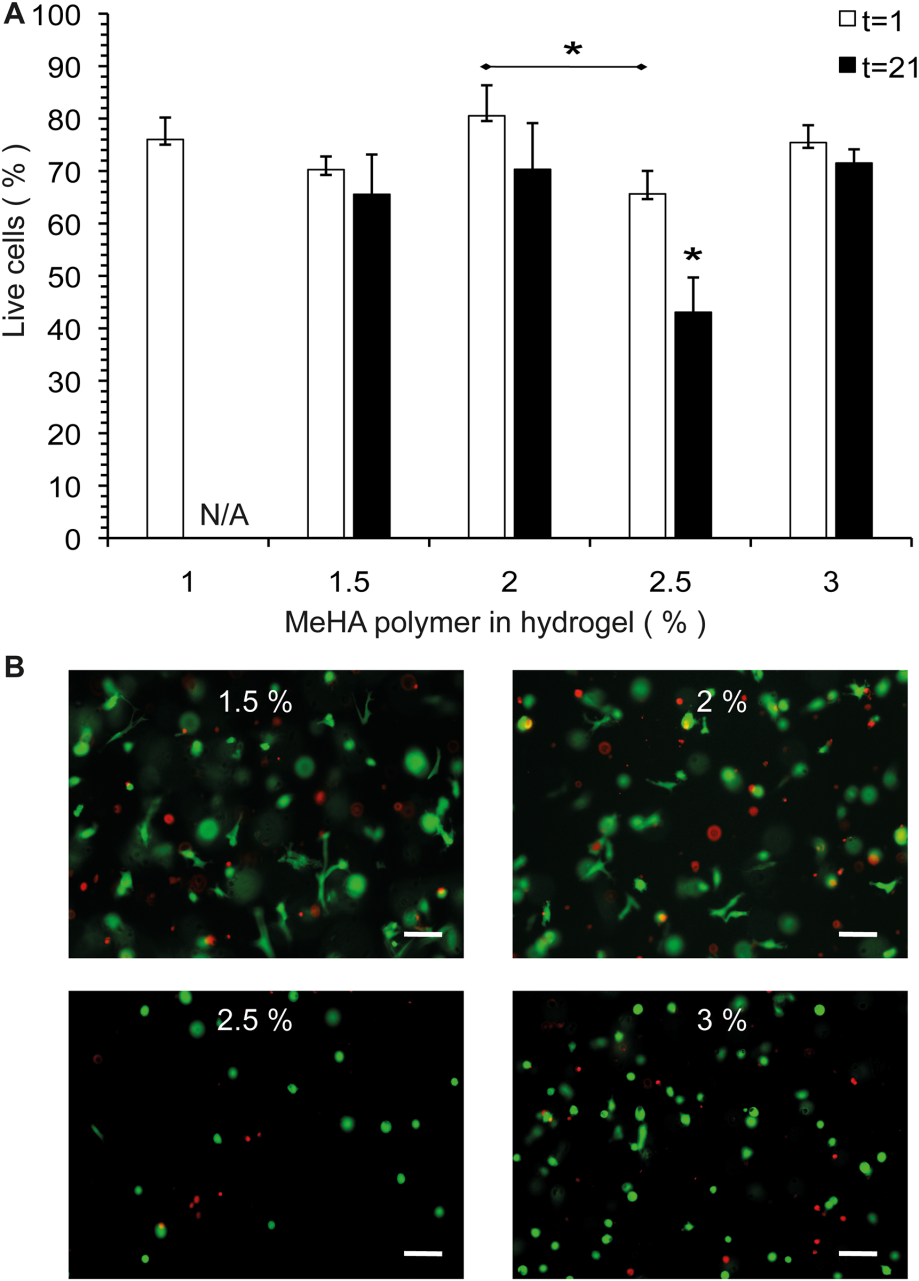
MSC survival and morphology in MeHA hydrogels. A. Survival of human MSCs after 1 and 21 days of MeHA hydrogel encapsulation. N/A: The 1% (w/v) hydrogels disintegrated before day 21. B. Cell morphology in the MeHA hydrogels after 21 days of culturing. Representative pictures are shown. Scale bars = 100 μm. * Represents *p*<0.05.

Survival of human bone marrow derived MSCs in MeHA hydrogels is presented in Fig 3. Average survival of MSCs in MeHA hydrogels after 1 day of gel encapsulation was 73.6±6.4%. When the live/dead analysis was repeated after 21 days of culturing, average MSC survival was still 64.4±12.2% (Fig 3A). The 1% (w/v) hydrogels disintegrated during culture in approximately 14 days; therefore data for day 21 are absent (N/A). Cell survival was significantly lower in the 2.5% (w/v) MeHA hydrogels compared to 2% (w/v) after 1 day. After 21 days of incubation cell survival in the 2.5% (w/v) gel was significantly lower compared to all other hydrogel concentrations. Also differences in cell morphology were observed: in the lower percentage (1.5–2% (w/v)) gels, many cells showed an elongated morphology within the gel (Fig 3B). At higher MeHA concentrations (2.5–3% (w/v)) gels, cells were only present in a round morphology.

### Osteogenic differentiation of MSCs in MeHA hydrogels

**Fig 4:**
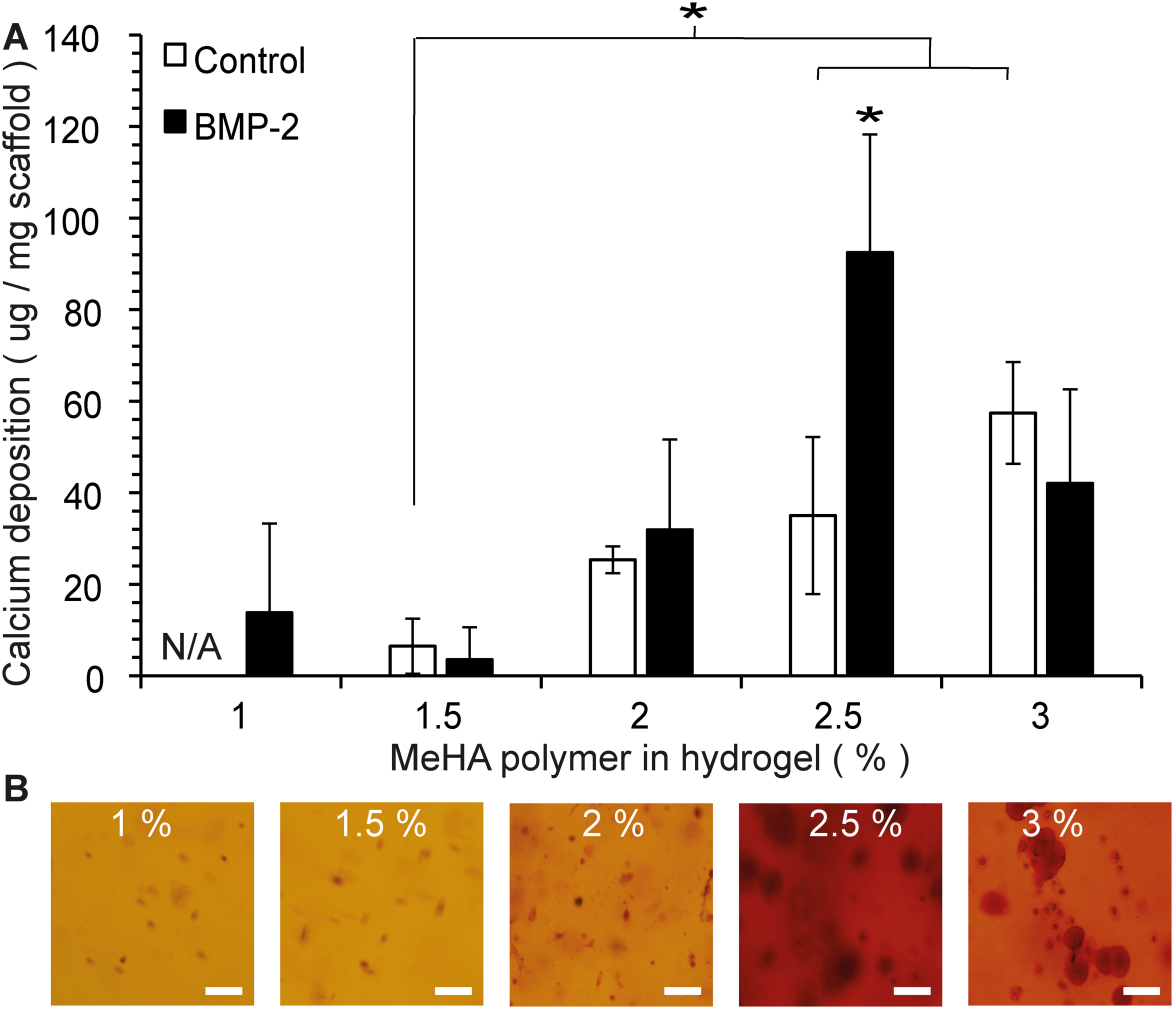
Osteogenic differentiation of MSCs in MeHA hydrogels. A. Calcium mineralization per mg of hydrogel scaffold after 21 days of culturing in αMEM (control, white bars) or αMEM supplemented with BMP-2 (black bars). Experiments performed in duplicate and replicated with 3 MSC donors. Data shown as mean ± SD. N/A: The 1% (w/v) hydrogels without BMP-2 disintegrated before the day 21 time point. * Represents *p*<0.05. B. Whole-mount Alizarin red staining of the MeHA hydrogels after 21 days of incubation in the presence of BMP-2. At lower MeHA concentration (1–1.5% (w/v)) gels, only some cells stained red. When the gel’s MeHA polymer concentration was increased, larger areas around the cells stained red, indicating calcium deposition into the surrounding matrix. Most intense staining is seen in the 2.5% (w/v) gels. Representative pictures are shown. Scale bars = 100 μm.

Osteogenic differentiation, measured by quantification of calcium deposition by MSCs is depicted in Fig 4. In hydrogels that did not receive an external osteogenic stimulus (BMP-2), increasing the MeHA polymer concentration led to significantly higher calcium precipitate formation (in 2.5–3% (w/v)) compared to 1.5% (w/v). Mineralization increased further when BMP-2 was added. Only in the 3% (w/v) group addition of BMP-2 did not lead to increased calcium deposition compared to control. Calcium deposition in the 2.5% (w/v) gel that received BMP-2 was significantly higher than in all other BMP-2 supplemented groups. Alizarin red staining confirmed the findings of the quantitative calcium assay, showing increased staining at higher polymer concentrations, which further increased when BMP-2 was added. The highest amount of mineralization was seen in the 2.5% (w/v) gels.

### 3D printability of MeHA hydrogels

**Fig 5:**
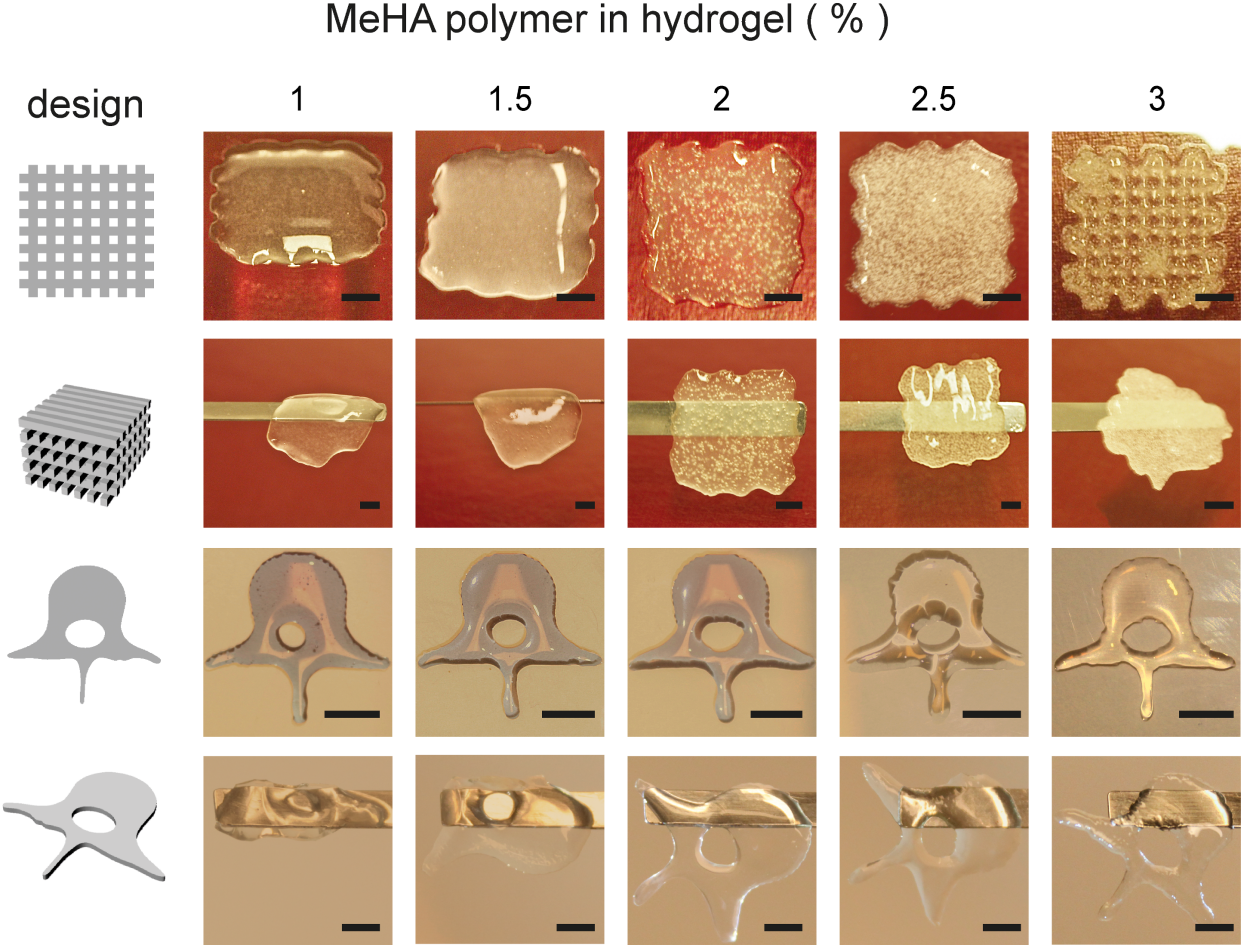
MeHA printability. Porous cubes (upper rows) and non-porous human L3 vertebrae shapes (lower rows) were 3D bioprinted, using the designs shown in the left column, and subsequently UV irradiated. After crosslinking, scaffolds were lifted using a spatula (horizontal bar) to test handling. Scale bars = 500 μm.

In order to assess the MeHA gel printability, two distinct designs were 3D printed, photocrosslinked and the resulting scaffolds were subsequently tested for handling with a spatula. (Fig 5). Hydrogels were printed in the form of a porous cube, and as a solid human lumbar vertebra (L3, scaled 1:10). In order to maintain porosity in scaffolds, 3% (w/v) MeHA was the only hydrogel rigid enough to enable crosslinking before collapsing under its own weight. In case of a solid scaffold design, the desired shape could be reached with gels ≥2% (w/v) and they could be handled well after crosslinking, indicating adequate network formation. In accordance to the results in the porous cubes, the ‘vertebral channel’ remained best intact in the 3% (w/v) gel, indicating that hydrogel rigidity also played a role in shape maintenance of these scaffolds. In order to maintain designed porosity at least 3% (w/v) hydrogel was needed, in non-porous structures scaffolds printed from ≥ 2% (w/v) hydrogels were functional.

### Overall assessment of optimal MeHA hydrogel properties

**Fig 6:**
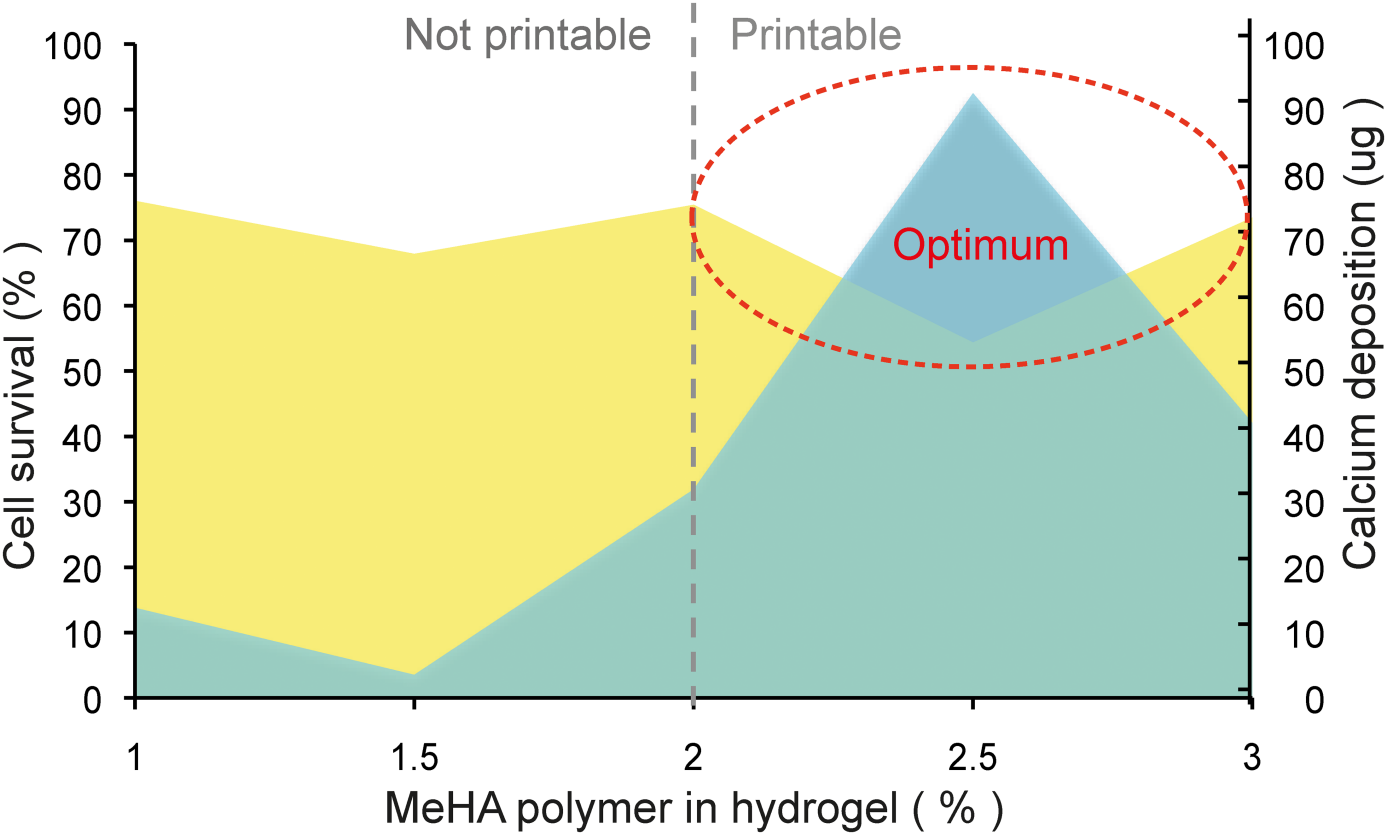
Optimal MeHA hydrogel selection. Combining MSC survival (yellow), osteogenic differentiation (green) and 3D printability (dotted grey line) data, reveals the boundary conditions for selection of optimal MeHA gel composition (dotted red line) for bone tissue engineering.

Taking together elastic properties, primary cell survival, MSC osteogenic differentiation and 3D bioprinting properties of the MeHA-polymer based hydrogels, the most suitable hydrogel concentration for different purposes in regenerative medicine could be defined. These properties are depicted in Fig 6, combining cell survival after 21 days of culture and osteogenic differentiation (mineralization), as a function of percentage of polymer dissolved, also indicating boundaries for 3D bioprinting requirements (both solid and porous). Based on these data, it becomes apparent that selection of the ideal hydrogel in this polymer concentration range, is highly dependent on the purpose of application in further experiments. The best suitable hydrogel for 3D bioprinting for bone applications is the 2.5% MeHA polymer.

## Discussion

In this study, high molecular weight hyaluronic acid was successfully methacrylated, thereby attaining photocrosslinking properties [25–27]. Methacrylation increased the final mechanical strength and this resulted in hyaluronic acid-based hydrogels that could be 3D bioprinted. Methacrylation has been applied in literature mainly on low molecular weight HA (<500 kDa) [28–30], that has less favorable biological properties [31]. Introduction of DMF as co-solvent in an aqueous reaction mixture, firstly described by Hachet *et al*. for HA with a molecular weight of 100 kDa [20], led to more efficient substitution than reactions performed in purely aqueous environment in their study. Following this approach, it was possible to obtain controlled DS on high molecular weight HA. The use of photopolymerizable methacrylate groups allowed mixing in of biological components (cells, bioactive molecules, etcetera [32]) at low rigidity, after which exposure at the appropriate wavelength of UV light led to rapid polymerization, causing minimal cell damage [17].

Hydrogels of different polymer concentrations were prepared and gel properties such as swelling, degradation rate and elastic moduli were dependent on the polymer concentration, and thereby tunable. MeHA gels showed good primary cell (human MSC) survival, for an extensive culture period of 3 weeks. When we compare this cell survival to survival in other hydrogels with tunable mechanical properties, such as polyethylene glycol (PEG), we observed much higher viability [33], and in contrast to the PEG, MeHA did not need addition of functional peptides (such as RGD) to enhance gel performance [34].

Most interestingly, the photopolymerized HA gel showed intrinsic osteogenicity depending on the gel concentration, even when no additional osteogenic stimulus was given in the medium. When gel rigidity increased, cell morphology changed from elongated to round cells, and although overall cell survival decreased, more osteogenic differentiation of MSCs was observed. This intrinsic osteogenicity of surviving cells has, to our knowledge, not been reported in literature. Changes in viscoelastic properties of MeHA gels have directly led to altered differentiation patterns of neural progenitor cells as reported in literature [35], indicating that mechanical properties of the material influence cell fate [36,37], which is in agreement with our findings. When an osteogenic stimulus (BMP-2) was added to the hydrogel with intrinsic osteogenicity, a synergistic pattern of increased mineralization was observed. These data indicate that the MeHA hydrogels can function as suitable scaffold material for bone regeneration purposes in regenerative medicine, when the appropriate hydrogel concentration and with this, visco-elastic properties are selected [38].

Printability of the MeHA hydrogels was thoroughly investigated, in a porous and non-porous (anatomical) scaffold design. 3D printability of a material allows smart scaffold design, for example, with 3D bioprinting specific deposition of (multiple) cell types, (local) presence of bioactive molecules, pore size and distribution, complex geometries and customized 3D shapes, according to individual needs [39] can be accomplished. Scaffold porosity is considered beneficial for bone regeneration as it lowers the diffusion distance within the constructs, allowing rapid tissue ingrowth and vascularization [40]. We have shown that 3D bioprinting applying a hydrogel with a relatively low elastic modulus is possible when solid bioprinting of the anatomically shaped scaffolds is needed. The open structure of the MeHA hydrogel together with its excellent biocompatibility result in good cell performance and may be favorable for various cell types.

We were, to our knowledge, the first to introduce primary cells to the resulting high molecular weight MeHA, induce (intrinsic) differentiation and apply this material in 3D bioprinted scaffolds. The high levels of cell survival and intrinsic osteogenic differentiation in a 3D bioprintable hydrogel are very promising for future applications of this material in regenerative medicine.

## Conclusion

By methacrylation of high molecular weight hyaluronic acid, 3D printable hydrogels were acquired. These hydrogels showed good primary cell survival and excellent spontaneous osteogenic differentiation *in vitro*. We defined boundary conditions for optimization of 3D bioprinted hydrogel based scaffolds for bone regeneration. MeHA-based hydrogels with intrinsic osteogenicity are promising scaffold materials for application in 3D printed, tissue engineered bone substitutes.
